# Menopause and Diabetes Risk Along with Trajectory of β-Cell Function and Insulin Sensitivity: A Community-Based Cohort Study

**DOI:** 10.3390/healthcare13091062

**Published:** 2025-05-05

**Authors:** Mi Jin Choi, Juyoun Yu

**Affiliations:** 1 College of Nursing, Gyeongsang National University, Jinju-si 52727, Republic of Korea; mot2345@gnu.ac.kr; 2 Department of Nursing, Changwon National University, Changwon-si 51140, Republic of Korea

**Keywords:** menopause, diabetes mellitus, type 2, insulin secretion, insulin resistance, glucose tolerance test

## Abstract

Background: The relationship between menopause and diabetes risk is unclear, with some studies indicating a weak association. This study examined changes in diabetes risk, β-cell function, and insulin sensitivity in relation to menopause. Methods: In this community-based cohort study, data from 6684 visits to 1224 women over a 16-year follow-up were analyzed. Diabetes risk changes were assessed in relation to the different menopausal phases: premenopausal (≥3 years before menopause), perimenopausal (2 years before to 1 year after menopause), and postmenopausal (≥2 years after menopause). Changes in β-cell function and insulin sensitivity indices were tracked, and their relationship with diabetes risk was assessed. Generalized estimating equations and linear mixed models were used, adjusting for covariates including age at menopause and obesity. Results: Diabetes incidence was 18.6% among participants. The odds ratio (OR) of diabetes increased by 1.03 times annually during the premenopausal period (OR 1.03; 95% CI 1.02–1.04) and decreased during the postmenopausal period (OR 0.96; 95% CI 0.95–0.97). The incident diabetes groups showed a decline in insulin sensitivity and β-cell function, resulting in a decrease in the disposition indices over time. A large change in insulin sensitivity, especially during the period immediately before the onset of diabetes, increased the risk of diabetes (OR 1.88; 95% CI 1.33–2.67). Conclusions: This study indicates an increased diabetes risk during the premenopausal periods, compared with that in the postmenopausal period, independent of age at menopause and obesity. Additionally, a decrease in insulin sensitivity followed by a subsequent decrease in β-cell function depending on the time of onset was related to the risk of diabetes. These findings enhance the understanding of diabetes risk and associated changes in insulin indices in relation to menopause, emphasizing the importance of health management and diabetes prevention for women in menopausal transition.

## 1. Introduction

Menopause is an important milestone for middle-aged women and is considered a significant transitional period that affects subsequent health states [1]. It is defined as the cessation of menstruation for 12 months, accompanied by a decrease in female sex hormones due to a decline in ovarian function [2]. The transitional changes in menopause last for several years before and after menopause [2]. The metabolic changes occurring in the period approximating menopause include central obesity associated with fat redistribution, relative androgen excess, and a subsequent increase in insulin resistance, which elevate the risk of metabolic syndrome and cardiovascular disease [3,4]. The relationship between metabolic changes and the development of diabetes supports the potential link between menopause and an increased risk of diabetes.

However, previous studies have reported conflicting results regarding the association between menopause and diabetes risk [5,6,7,8,9]. Some studies have reported no difference in the risk of diabetes and impaired fasting glucose before and after menopause [10], suggesting that the steady rise in the risk of diabetes after menopause is difficult to attribute to menopause itself and is rather a consequence of aging [7]. Conversely, others have reported that the risk of diabetes is related to menopausal age, reproductive lifetime, or the levels of female hormones during menopause [11,12,13,14]. Despite the evidence linking female hormone deficiency to an increased risk of diabetes, inconsistencies in the results of various studies may be attributed to limitations in study design or methodology. These may include the inadequate tracking of changes during the menopausal transition, variations in the methods of assessing diabetes or its risk, and the influence of confounding variables such as aging and obesity. Therefore, more robust methodologies and longitudinal data are required to clarify these inconsistencies.

Despite the critical role of β-cell function and insulin sensitivity in the pathophysiology of type 2 diabetes, studies that have accurately evaluated β-cell function and insulin sensitivity during menopause remain limited [15]. Type 2 diabetes is caused by a progressive decline in β-cell insulin secretion due to various environmental and genetic factors, frequently occurring in the context of insulin resistance [16]. Previous research has confirmed that diabetes occurs when decreased β-cell function fails to compensate for increased insulin resistance among men and women aged 40–69 years [17]. Therefore, there is a clear gap in the understanding of the trajectory of β-cell function and insulin sensitivity over time, particularly across the phases of menopause in women and the association with diabetes onset. Investigating these trajectories and their impact on diabetes risk during the menopausal transition could facilitate a deeper understanding of the pathophysiology of diabetes.

Therefore, a well-designed study that uses longitudinal data to elucidate the changes in diabetes risk around the menopausal period and validate the association between diabetes incidence and risk through reliable and valid methods with adequate control for confounding variables is warranted. Such a study could enhance our understanding of the unexplored aspects of the relationship between menopause and diabetes. Therefore, in this community-based cohort study, we tracked and analyzed the following: (1) the risk of diabetes through the different phases of menopausal transition, (2) the trajectory of β-cell function and insulin sensitivity based on the onset and timing of diabetes, and (3) the baseline level and changes in β-cell function and insulin sensitivity with respect to the menopausal phase, and their association with the risk of diabetes.

## 2. Materials and Methods

We used data collected over 16 years of follow-up from women in the menopausal transition phase. The incidence of diabetes was confirmed using oral glucose tolerance tests (OGTTs) and glycated hemoglobin A1c (HbA1c). Additionally, we assessed β-cell function and insulin sensitivity using indices derived from OGTTs while adjusting for age at menopause, obesity, and other confounding variables.

### 2.1. Study Participants

This study targeted women who had reached menopause during the follow-up period, allowing us to determine whether they developed diabetes. Participants were recruited from a community-based cohort (the Ansan and Ansung cohorts), which is part of the Korean Genome and Epidemiology Study (KoGES). The KoGES is a large prospective cohort study devised to identify the genetic and environmental risk factors for major chronic diseases, whose detailed profile has been described elsewhere [18]. The Ansan and Ansung cohorts recruited men and women between the ages of 40 and 69 years. Follow-up was conducted at 2-year intervals commencing in 2001, and, to date,, the 10th follow-up has been completed [18,19]. The current study obtained data from 33,997 visits of 5272 women up to the 8th follow-up completed in 2018.

**Figure 1:**
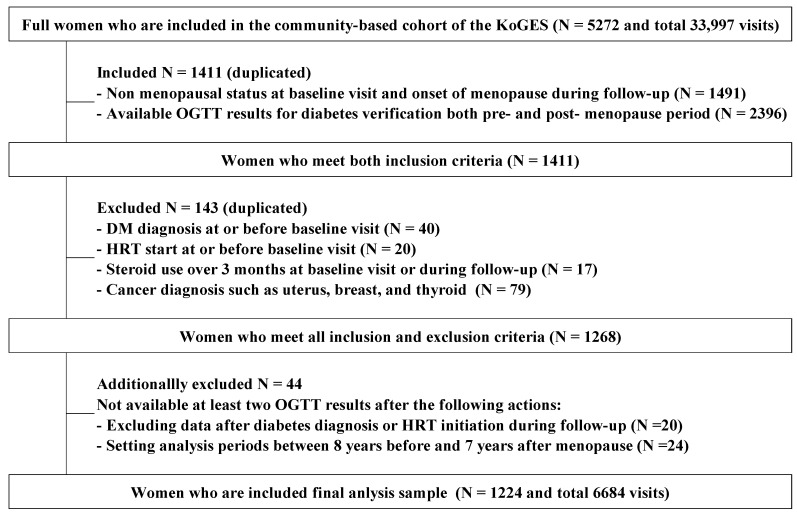
Diagrammatic representation of the selection of the analysis sample. From the 5272 women in the Ansan and Ansung cohorts of the Korean Genome and Epidemiology Study (KoGES), those who reached menopause during follow-up and met the criteria for tracking diabetes risk before and after menopause were selected. Inclusion criteria involved the following: (1) women without menopause at baseline who reached menopause during follow-up, allowing the confirmation of menopausal age; (2) the availability of at least two OGTT results around menopause. Exclusions applied to those undergoing hormone replacement therapy, with a diabetes diagnosis at baseline, prolonged steroid use, or certain cancer diagnoses. Additionally, women with fewer than two OGTT results remaining after excluding data due to diabetes diagnosis, HRT initiation, or within the 16-year analysis period were omitted. Ultimately, 6684 visit data from 1224 women were analyzed, with an average follow-up of 5.5 times per individual. Note: (1) There were no cases of premature menopause (<40 years old) because the original KoGES cohort recruited women between the ages of 40 and 69 years, and this study only included women who experienced menopause during the follow-up period. (2) The data of women who were diagnosed with diabetes or initiated HRT were censored at the time of the first diagnosis or initiation. Therefore, data corresponding to 1023 visits were excluded from the analysis. KoGES, Korean Genome and Epidemiology Study; OGTT, oral glucose tolerance test; HRT, hormone replacement therapy.

Considering that this study aimed to track the risk of incident diabetes before and after menopause, the inclusion criteria were as follows: (1) women who did not have menopause at the baseline visit and reached menopause during the follow-up period, such that the menopausal age could be confirmed, and (2) women whose OGTT results around menopause were available at least twice, to facilitate the tracking of diabetes, β-cell function, and insulin sensitivity (Figure 1). The reason for establishing the criterion of having more than two measurements per individual is to adequately capture changes over time, as well as individual variability or correlation. Among the 1411 women who met the inclusion criteria, 143 were excluded for the following reasons: (1) past or present history of hormone replacement therapy (HRT) at the baseline visit, (2) meeting the diagnostic criteria for diabetes at the baseline visit, (3) steroid use for more than 3 months, or (4) diagnoses of uterine, breast, or thyroid cancers. Women with premature menopause (before 40 years of age) were not included because the original cohort enrolled women older than 40 years, and this study included only woman who had not reached menopause at the baseline visit. In the event of a diagnosis of diabetes or the initiation of HRT during follow-up, data from that point were excluded (1023 data points were deleted). Forty-four women with fewer than two OGTT results after data deletion due to the diagnosis of diabetes, HRT initiation, or within the 16-year data analysis period were excluded from the analysis sample. Finally, 6684 data points from 1224 women (from June 2001 to November 2018) were included in the analysis (Figure 1). Overall, 1224 women were followed up for an average of 5.5 times [standard deviation (SD): 1.8], with participation rates from visit 2 to visit 9 being 94%, 88%, 84%, 86%, 86%, 85%, 89%, and 88%, respectively. Among the 1224 women, 228 (18.6%) developed incident diabetes during a median observation period of 15.7 years (interquartile range, 14.3–15.9; range, 1.6–16.6).

### 2.2. Procedures and Variables

Participants were scheduled to visit the research institutions every 2 years for a survey and examination. The survey was conducted by trained examiners using a computer-assisted personal interview system, and anthropometric and laboratory measurements were performed using standardized protocols [18,19,20]. The research institutions—medical institutions and health-screening centers—were responsible for recruiting participants, obtaining consent, collecting data and resources, and transferring them to the National Institute of Health (NIH). The NIH, an institute affiliated with the Korea Disease Control and Prevention Agency, oversaw the entire cohort study and managed the quality of performance and data, including in-depth data purification [18,19,20].

The survey items included general information, such as age, education, marital status, income, disease and treatment history, and family history of diabetes; lifestyle factors, such as smoking, alcohol consumption, and physical activity; and gynecological history, including age at menarche and menopause. Anthropometric measurements such as height, weight, and waist circumference were performed. Laboratory sampling was conducted with overnight fasting for at least 8 h to assess HbA1c, total cholesterol (TC), high-density lipoprotein (HDL) cholesterol, and triglycerides (TGs). At each visit, an OGTT was conducted using a 75 g glucose load, and plasma samples were collected at 0, 60, and 120 min post-load to measure glucose and insulin levels. The samples were analyzed by a qualified central laboratory using standardized methods [17,20]. Plasma glucose was quantified using the hexokinase enzymatic method, while plasma insulin concentrations were determined through radioimmunoassay. HbA1c was measured using high-performance liquid chromatography.

To confirm menopause at each visit, the participants were asked about the cessation of menstruation for the past 12 months. When menopause was confirmed, menopausal age, defined as the age at the last menstrual period, and female hormone use after menopause were investigated. Because each woman’s menopausal age varied during the 16-year follow-up period, we set each woman’s menopausal age as 0 and aligned each visit according to the difference between the age at each visit and the age at menopause (in years). Considering the purpose of this study, the small number of visits with large standard errors at the extremes, and the biennial visit cycle of the original cohort, the period from 8 years before menopause to 7 years after menopause was divided into 2-year cycles (eight intervals over 16 years: −8 to −7, −6 to −5, −4 to −3, −2 to −1, 0 to +1, +2 to +3, +4 to +5, and +6 to +7). Additionally, to compare the degree of change during the menopausal transition, the period was divided into three phases based on the timing of the change in estradiol around menopause and our observation intervals [2]: (1) premenopausal period, 3 to 8 years before menopause; (2) perimenopausal period, 2 years before to 1 year after menopause; (3) postmenopausal period, 2 to 7 years after menopause. As an exception, “perimenopausal incidence of diabetes” included the confirmation of diabetes at a visit at 2 or 3 years after menopause, accounting for the potential delay in the detection of diabetes due to the 2-year visit cycle.

The verification of incident diabetes was solely based on laboratory results obtained at each visit. Diabetes was diagnosed when fasting plasma glucose was ≥126 mg/dL, 2 h plasma glucose at a 75 g glucose OGTT was ≥200 mg/dL, or HbA1c was ≥6.5%, according to the diagnostic criteria established by the American Diabetes Association [21]. Pancreatic β-cell function was evaluated by calculating the 60 min insulinogenic index (IGI_60_) and homeostasis model assessment of the β-cell function (HOMA-β) index [17,21,22] as follows: IGI_60_ = [60 min insulin − 0 min insulin (µU/mL)]/[60 min glucose − 0 min glucose (mmol/L)]; HOMA-β = [fasting insulin (µU/mL) × 20]/[fasting glucose (mmol/L) − 3.5]. The composite (Matsuda) insulin sensitivity index (ISI) and homeostasis model assessment of insulin resistance (HOMA-IR) index were used to evaluate insulin sensitivity or resistance [17,22,23,24] as follows: composite ISI = 10,000/$\sqrt{\left(fasting\;glucose\times fasting\;insulin)\times (mean\;glucose\times mean\;insulin\right)}$ [glucose, mg/dL; insulin, µU/mL; mean values derived using 0, 60, 120 min values of the OGTT]; HOMA-IR = [fasting glucose (mmol/L) × fasting insulin (µU/mL)]/22.5. Two disposition indices (DIs) were also calculated using the IGI and composite ISI and HOMA-β and HOMA-IR [17,25] as follows: DI_IGI, ISI_ = IGI_60_ × composite ISI; DI_HOMA-β, HOMA-IR_ = HOMA-β/HOMA-IR.

The reproductive lifetime was calculated by subtracting the age at menarche from the age at menopause. The body mass index (BMI) was calculated by dividing the body weight (kg) by the square of height (m^2^). The following were included as categorical variables: study site (Ansan or Ansung), education (less than 7 years, 7–12 years, or more than 12 years), marital status (single, married, or divorced/separated/bereaved), income (less than KRW 1 million, KRW 1 to 3 million, or more than KRW 3 million), family history of diabetes (“yes” if any parents, siblings, or offspring had diabetes), history of gestational diabetes (“yes” for a diagnosis of gestational diabetes), hypertension (“yes” for a diagnosis of hypertension), smoking (never/former or current), exercise (none, one or two times per week, or three times or more per week), and alcohol consumption (none, less than 20 g per day, or 20 g or more per day). For adjustment during the analysis, study site, education, marriage, income, family history of diabetes, history of gestational diabetes, hypertension, and age at menopause were used as time-invariant variables, while BMI, waist circumference, HDL, TG, smoking, exercise, and alcohol consumption were used as time-variant variables.

### 2.3. Data Analysis

Descriptive statistics were calculated for the main variables, including frequencies (percentages) for categorical variables and means (standard deviations) for continuous variables. The ANOVA and chi-squared test were used to analyze group differences, with post hoc tests when necessary. Generalized estimating equation (GEE) models were applied to estimate the risk of diabetes by accounting for correlation within individuals, which is crucial for repeated measures data, allowing for robust estimation while adjusting for covariates [26,27]. To assess the effect of each menopausal phase (pre-, peri-, and postmenopausal), a model including three phases of aging (pre-, peri-, and postmenopausal aging) was used. This model structure enabled us to specifically evaluate changes over distinct menopausal phases, providing insight into how these transitions impact diabetes risk. Another sequential analysis was conducted to examine the relationship between diabetes risk and baseline or changes in β-cell functions and insulin sensitivity, after adjusting for the effects of age, obesity, and other covariates. Baseline age was adjusted in model 1; BMI and waist circumference were added to model 2; and finally, other covariates were added to model 3. The risk of diabetes was presented using odds ratios (ORs) and corresponding 95% confidence intervals (CIs). Linear mixed models (LMMs) were chosen to analyze changes in β-cell functions and insulin sensitivity around menopause because they account for both fixed and random effects, capturing individual variability and allowing for the inclusion of time-varying covariates [28]. The estimated means for each β-cell function or insulin sensitivity index over time were calculated for each group according to the timing of diabetes incidence using the LMM with random effects of individuals and adjustments for covariates. All β-cell function and sensitivity indices were log-transformed for analysis and back-transformed for presentation. The covariates included in multiple models were age at menopause, BMI, waist circumference, HDL, TG, study site, education, marital status, income, family history of diabetes, hypertension, smoking, exercise, and alcohol consumption. The level of significance was set at *p* < 0.05. All data analyses were performed using the SPSS Modeler and SPSS (version 22.0, IBM Corp., Armonk, NY, USA).

## 3. Results

Among 1224 women without menopause and diabetes at the baseline visit, 228 (18.6%) developed diabetes during a maximum follow-up period of 16.6 years (median 15.7 years). The distribution of diabetes incidence with respect to the period of menopause was as follows: 36 (2.9%) women developed diabetes between 3 and 8 years before menopause (premenopausal incident diabetes), 77 (6.3%) women developed diabetes between 2 years before and 3 years after menopause (perimenopausal incident diabetes), and 115 (9.4%) women developed diabetes between 4 and 7 years after menopause (postmenopausal incident diabetes). Table 1 presents the baseline characteristics of the four study groups, stratified by diabetes onset and non-progression. Women with premenopausal incident diabetes had a lower baseline age (*p* = 0.001), higher menopausal age (*p* = 0.007), and a longer reproductive lifetime (*p* = 0.006), compared with the women with postmenopausal incident diabetes (Table 1). Women with perimenopausal or postmenopausal incident diabetes had higher BMI, waist circumference, and TG levels, lower HDL levels, and a higher frequency of hypertension, compared with women with non-progression to diabetes (*p* < 0.001). A family history of diabetes (*p* = 0.004) and alcohol consumption of 20 g or more per day (*p* = 0.003) were more common in women with premenopausal or perimenopausal incident diabetes, compared with women who did not develop diabetes. The proportion of current smokers (*p* = 0.003) was higher in women with premenopausal incident diabetes, compared with women who did not develop diabetes. All women with incident diabetes had higher baseline fasting plasma glucose (*p* < 0.001) and HbA1c levels (*p* < 0.001), lower composite ISI values (*p* < 0.001), and lower DIs (*p* < 0.001). These baseline conditions suggest that women who develop diabetes later in life are more susceptible to diabetes-related risk factors, including impaired β-cell function and reduced insulin sensitivity.

**Figure 2:**
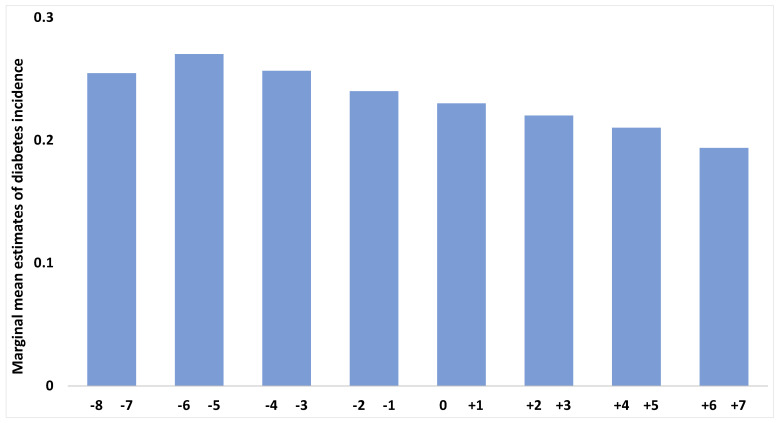
Mean estimates of diabetes incidence in the years before or after menopause. Mean estimates of diabetes incidence were illustrated from 8 years before to 7 years after menopause, divided into 2-year intervals (eight intervals over 16 years: −8 to −7, −6 to −5, −4 to −3, −2 to −1, 0 to +1, +2 to +3, +4 to +5, and +6 to +7). A marginal model with a generalized estimating equation was used to estimate the risk of diabetes, accounting for within-individual correlation and adjusting for covariates such as age at menopause, study site, education, marital status, income, family history of diabetes, hypertension, smoking, exercise, alcohol consumption, BMI, waist circumference, HDL, and TG [covariate values fixed in the model: age at menopause = 51.1 years; BMI = log 3.18 (24.2 kg/m^2^); waist circumference = log 4.37 (79.3 cm); HDL = log 3.86 (47.7 mg/dL); TG = log 4.64 (103.5 mg/dL)].

We investigated changes in the risk of developing diabetes by categorizing the menopausal transition period into three phases based on the menopausal year (premenopausal period = 3 years and more before menopause, perimenopausal period = 2 years before to 1 year after menopause, and postmenopausal period = 2 years and more after menopause). The ORs of diabetes among these three periods were calculated using the GEE method, with adjustments for age at menopause, BMI, waist circumference, HDL cholesterol, TGs, study site, education, marital status, income, family history of diabetes, hypertension, smoking, exercise, and alcohol consumption. In the premenopausal period, the ORs of diabetes rose slightly by 1.03 times per year (OR 1.03; 95% CI 1.02–1.04; *p* < 0.001), which was higher than that in the other periods (Figure 2; Table 2). The ORs of diabetes during the perimenopausal period were maintained, and there was no significant change with aging (OR 1.00; 95% CI 0.99–1.01; *p* = 0.862). Finally, the ORs for diabetes in the postmenopausal period decreased with aging (OR 0.96; 95% CI 0.95–0.97; *p* < 0.001). Therefore, diabetes risk varied across the three periods.

**Figure 3:**
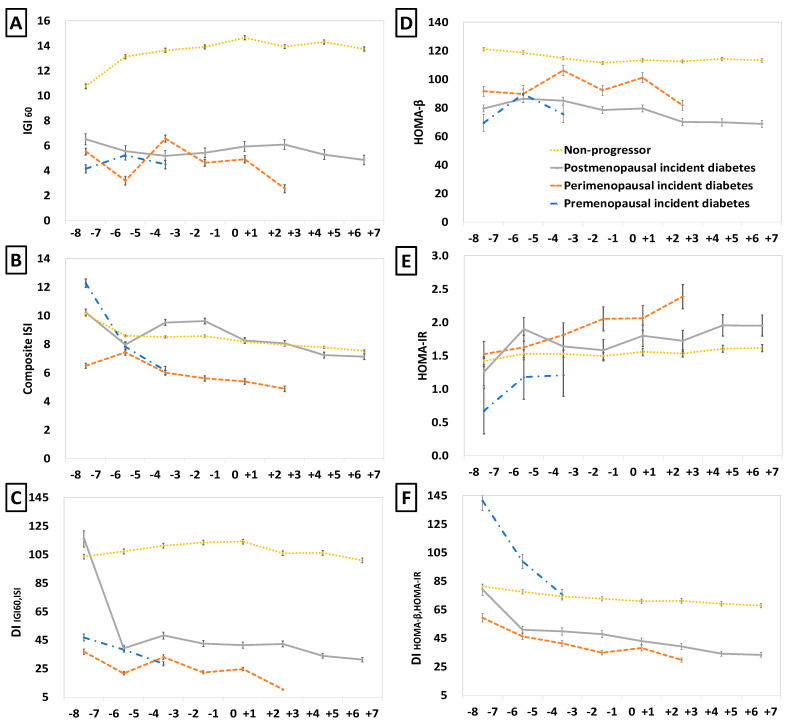
Changes in β-cell function and insulin sensitivity in the years before and after menopause. Changes in β-cell function and insulin sensitivity/resistance indices were estimated over the period from 8 years before to 7 years after menopause across four groups, categorized by diabetes onset: premenopausal, perimenopausal, postmenopausal incident diabetes, and non-progressors. The indices analyzed included (**A**) IGI_60_ = 60 min insulinogenic index; (**B**) composite ISI = composite insulin sensitivity index; (**C**) DI _IGI60, ISI_ = disposition index using IGI_60_ and ISI; (**D**) HOMA-β = homeostasis model assessment of β-cell function index; (**E**) HOMA-IR = homeostasis model assessment of insulin resistance index; (**F**) DI_HOMA-β, HOMA-IR_ = disposition index calculated using the HOMA-β and HOMA-IR. Linear mixed models were employed to calculate estimated means, incorporating the random effects of individuals and adjustments for covariates, including age at menopause, BMI, waist circumference, HDL, TG, study site, education, marital status, income, family history of diabetes, hypertension, smoking, exercise, and alcohol consumption. All indices were log-transformed for analysis and back-transformed for presentation. Note: Negative values for the IGI_60_ and DI _IGI60, ISI_ (721 data points; 11%) and data after diabetes diagnosis and the start of HRT (1023 data points, 15%) were excluded from the analysis. The Non-progressor group is represented by yellow, the premenopausal incident diabetes group by blue, the perimenopausal incident diabetes group by orange, and the postmenopausal incident diabetes group by gray.

To track changes in β-cell function and insulin sensitivity or resistance over time, we analyzed the IGI_60_, HOMA-β, composite ISI, HOMA-IR, and the two DIs in the four groups (Figure 3). The three incident diabetes groups showed a decline in composite ISI, an elevation in HOMA-IR, and a decline in the IGI_60_/HOMA-β with a fluctuation, resulting in a gradual decrease in the DIs over time. During the premenopausal period, the perimenopausal incident diabetes group experienced a steady decrease in the composite ISI and an increase in HOMA-IR, while the postmenopausal incident diabetes group exhibited these changes prominently during the perimenopausal period. In the perimenopausal period, the IGI_60_/HOMA-β change declined with a fluctuation in the perimenopausal incident diabetes group, while these values were maintained or showed a slight increase in the postmenopausal incident diabetes group. During the postmenopausal period, the non-progression and postmenopausal incident diabetes groups showed similar levels of the composite ISI with slight differences in HOMA-IR, whereas IGI_60_/HOMA-β and disposition indices showed large differences and were significantly lower in the postmenopausal incident diabetes group. Conversely, the non-progression group showed an increase in IGI_60_ with a slight decrease in the composite ISI and stable levels of HOMA-IR and HOMA-β over time, consequently maintaining high and stable levels of DIs. This stable pattern observed in the non-progression group across all the periods indicates that proper β-cell function corresponds to insulin sensitivity or resistance. In contrast, the diabetes groups exhibited decreased β-cell function, which failed to adequately compensate for reduced insulin sensitivity or increased insulin resistance.

We analyzed the ORs of diabetes along with baseline levels and changes in the IGI_60_, composite ISI, and DI during the peri- and postmenopausal periods to examine the relationship between changes in β-cell function/insulin sensitivity and the risk of diabetes according to the menopausal period and diabetes groups (Table 3). Notably, both the baseline level of the DI and changes in the DI during the pre-, peri-, and postmenopausal periods were statistically significant predictors of diabetes in the peri- and postmenopausal incident diabetes groups. For example, a high baseline level of the DI lowered the risk of postmenopausal incident diabetes (OR 0.82; 95% CI 0.72–0.93; *p* = 0.003), and a large change in the DI in each period increased the risk of diabetes (premenopausal period: OR 1.17, 95% CI 1.06–1.30, *p* = 0.003; perimenopausal period: OR 1.17, 95% CI 1.04–1.32, *p* = 0.011; postmenopausal period: OR 1.14, 95% CI 1.06–1.24, *p* = 0.001). A large change in ISI, especially during the period immediately before the onset of diabetes (e.g., premenopausal period for the perimenopausal incident diabetes group and the perimenopausal period for the postmenopausal incident diabetes group), increased the risk of diabetes (OR 1.88; 95% CI 1.33–2.67; *p* < 0.001), and a high baseline ISI lowered the risk of diabetes (OR 0.54; 95% CI 0.42–0.70; *p* < 0.001). The baseline value and change in IGI showed a weaker relationship with the risk of diabetes, compared with the other indices. However, the ORs for some baseline levels and changes in IGI attained statistical significance after adjusting for obesity and other variables. Comparing the change in the ORs of diabetes with additional covariate adjustments revealed more significant indices after adjusting for baseline age (model 1), baseline BMI, waist circumference (model 2), and other variables (model 3).

## 4. Discussion

This study investigated changes in the risk of diabetes and the trajectory of β-cell function and insulin sensitivity during the pre-, peri-, and postmenopausal periods using longitudinal data collected over 16 years, considering the timing of incident diabetes and confounding variables. Our results showed that the OR of developing diabetes gradually increased during the premenopausal period, was sustained during menopause, and decreased during the postmenopausal period. Moreover, women with incident diabetes exhibited a decrease in the composite ISI before the onset of diabetes, followed by a subsequent decrease in IGI_60_ and DIs. Low baseline levels and large changes in β-cell function and insulin sensitivity indices increased the risk of diabetes. These findings confirmed that the risk of diabetes, along with related β-cell function and insulin sensitivity around menopause, underwent differential alterations over time, depending on the timing of diabetes onset.

This study identified differences in the risk of diabetes depending on the period relative to menopause. While our findings show an elevated risk of diabetes during the premenopausal period and a decreased risk during the postmenopausal period, previous studies have reported conflicting results regarding the link between menopause and diabetes risk, leading to ambiguity in their relationship [5,6,7]. Some studies reported that early menopausal age was associated with a higher risk of diabetes. One study showed a 32% greater risk of diabetes with menopause before the age of 40 years, while another showed a 20% greater risk with menopause before the age of 45 [12,13]. In addition, a shorter reproductive lifetime, lower premenopausal estradiol levels, and slower change in follicle-stimulating hormone levels before menopause were also related to a higher risk of diabetes [14,29,30]. This suggests that the magnitude and duration of female hormone deficiency related to menopause may be associated with a higher risk of diabetes. Our findings align with studies indicating hormonal changes as significant factors in diabetes risk, as they highlight the role of β-cell function and insulin sensitivity. However, studies examining the relationship between menopause and diabetes risk should exercise caution when addressing aging and obesity, which are common conditions in women around the menopausal period and strong risk factors for diabetes. A previous study reported that the significance of the relationship between menopause and diabetes risk disappeared after adjusting for age [31]. Some argued that the increased risk of diabetes during menopause was not due to menopause itself but rather other diabetes-related risk factors, such as increased age and accompanying obesity, because the increase in the risk of diabetes with respect to menopause changes steadily over time rather than abruptly [5]. Our approach considered these factors by adjusting for age at menopause, obesity, and other covariates, confirming that the increased risk is indeed related to the physiological changes occurring during the menopausal transition. Furthermore, we tracked the changes in the risk of diabetes and the trajectory of β-cell function and insulin sensitivity over the period of menopausal transition using longitudinal data, while considering the changes and correlations for each woman by verifying diabetes solely with laboratory findings. By sequentially adding baseline age, BMI, waist circumference, and other variables to the models, we identified the relationship between the risk of diabetes and changes in β-cell function and insulin sensitivity, independent of age and obesity, providing a more comprehensive understanding that supports and extends the existing literature.

We identified an elevated risk of diabetes during the premenopausal period and a decreased risk during the postmenopausal period. This may be linked to increases in fat mass observed during these periods, in addition to direct hormonal changes. Greendale et al. (2019) found that accelerated fat mass, particularly central adiposity, persisted from the onset of the menopausal transition to 2 years after the final menstrual period, after which it stabilized [32]. These changes align with the elevated diabetes risk and increased insulin resistance observed in our study, as fat accumulation is known to heighten insulin resistance and diabetes risk. We hypothesize that women with a predisposition to diabetes may face a higher risk of developing the disease due to increased insulin resistance from fat accumulation and inadequate β-cell compensation during the premenopausal phase [32,33,34]. Moreover, in this study, BMI and waist circumference were very strong factors for the incidence of diabetes, even among participants who were not highly obese. Previous research has indicated that South Asians may face an increased likelihood of diabetes due to diminished β-cell function, even if their BMI is not high [35,36]. Given that this study utilized longitudinal data and that the GEE model accounts for intra-individual correlation rather than isolated measurements, it can be interpreted that even if participants’ BMI or waist circumference is not high, an increase over time indicates a significantly higher risk of developing diabetes. Notably, the diabetes incidence groups exhibited lower β-cell function compared to the non-incidence group in our study. Our findings may suggest that, beyond baseline obesity, changes in obesity-related factors over time and the deterioration of β-cell function could play crucial roles in diabetes onset, highlighting the value of conducting longitudinal assessments. Further research should investigate the link between changes in fat accumulation and diabetes onset, considering predispositions to diabetes in women undergoing menopause transition.

This study tracked changes in β-cell function and insulin sensitivity over time in the period approximating menopause. Studies that have assessed the association between menopause and insulin secretion or insulin sensitivity are scarce [37,38,39]. Studies comparing insulin sensitivity in premenopausal and postmenopausal women have reported no differences between the two groups [37,38]. Walton et al. observed a 50% decrease in insulin secretion in postmenopausal women, compared with that in premenopausal women, which was accompanied by a 50% rise in insulin sensitivity, a low insulin elimination rate, and subsequently, similar plasma insulin concentrations [39]. These results may imply that simple comparisons of premenopausal and postmenopausal women or simple measurement methods that do not consider insulin dynamics are not equipped to detect insulin changes related to menopause. Our study tracked and compared changes in indices representing β-cell function and insulin sensitivity through periods of menopausal transition in four groups according to the onset and timing of incident diabetes. This is because we hypothesized that the patterns of changes occurring through the menopausal periods may differ among women with different predispositions toward diabetes, as evidenced by the differences in baseline characteristics among groups. According to our results, the groups with different timelines of diabetes onset exhibited differential patterns of changes in β-cell function and insulin sensitivity relative to the menopausal period, which differed from the stable pattern over the entire period in women who did not develop diabetes. The changes in the composite ISI and HOMA-IR were noticeable in all the diabetes groups immediately before the onset of diabetes during the menopausal period. During the perimenopausal period, the changes in the IGI_60_, HOMA-β, and DIs varied according to the diabetes group: the perimenopausal incident diabetes group showed a decrease in these parameters with fluctuation, while the postmenopausal incident diabetes group showed a slight increase or maintenance in their values. Ohn and Kwak et al. confirmed that impaired β-cell compensation for decreased insulin sensitivity is critical for the development of diabetes, reporting a decrease in the composite ISI and no compensatory increase in the IGI_60_ in diabetes progressors throughout a 10-year follow-up period [17]. The pattern of changes in β-cell function and insulin sensitivity over the menopausal transition period observed in this study also supports the fact that decreased insulin sensitivity (or increased insulin resistance) and the compensatory failure of β-cell function are associated with the development of diabetes.

In addition, we examined the baseline level and changes in β-cell function, insulin sensitivity indices, and the associated risk of diabetes during the period around menopause. While IGI_60_ showed a weak relationship with the risk of diabetes, lower baseline levels and larger changes in the composite ISI and DI significantly increased the risk of diabetes. The DI at baseline and changes during the pre-, peri-, and postmenopausal periods showed a consistently significant relationship with the risk of diabetes. These results suggest that the ability to successfully respond to changes in insulin sensitivity (or resistance) represented by DI, rather than the insulin secretion ability itself (represented by the IGI), may be directly related to the risk of diabetes. Therefore, the DI could be a more stable and suitable indicator of the risk of diabetes [25,40].

This study had some methodological advantages. First, longitudinal data accrued during a 16-year follow-up of a well-designed and managed cohort were used to track and compare changes related to menopause. Time-varying variables (BMI, waist circumference, HDL, TGs, smoking, alcohol consumption, and exercise) were subjected to statistical analysis to determine the correlation within individuals. Second, the occurrence of diabetes was verified using a reliable method (fasting or 2 h plasma glucose derived from the OGTT or HbA1c) at each visit, and the risk of diabetes and changes in β-cell function and insulin sensitivity indices were tracked together in consideration of the onset and timing of diabetes. Lastly, we tried to minimize the effects of confounding factors: women with factors affecting the menopausal status and diabetes risk, such as the use of HRT or steroids, were excluded. If HRT was initiated or diabetes was diagnosed during follow-up, data from that time point were excluded, and a statistical analysis was performed by adjusting for age at menopause, obesity, and other confounding variables.

This study had some limitations in its design because it used the KoGES cohort data from a 2-year visit schedule, which was designed to identify chronic disease risk, not the relationship between menopause and the risk of diabetes. Specifically, because the analysis period was not established according to the date of the final menstrual period but was based on the menopausal age confirmed at each visit, it may be difficult to calculate the precise period, and some discrepancies may exist compared with the actual period. Furthermore, there may have been a time delay between the actual onset of diabetes and diagnosis due to the 2-year visit cycle, which caused the diagnosis period for perimenopausal incident diabetes to be set to up to 3 years after menopause. Such biennial data collection may have resulted in an underestimation of diabetes incidence during specific menopausal transitions, as intermittent data points might miss the exact timing of diabetes onset. These limitations highlight the need for more frequent data collection in future studies to better capture the precise temporal relationships between menopause and diabetes risk. Moreover, this study could not utilize the parameters that were not included in the KoGES dataset, such as LDL-C, which could be relevant to the understanding of diabetes risk. It is important to note that in this study, diabetes was defined according to the ADA criteria as meeting one of the following thresholds: FPG, 2 h PG, or HbA1c [16]. HbA1c can be influenced by other factors, which may affect study results, necessitating careful interpretation [16,41]. Among the 228 incident cases of diabetes identified in this study, 107 cases (46.9%) met both the HbA1c and glucose criteria, whereas 35 cases (15.4%) were diagnosed based solely on the HbA1c criterion. In instances of discrepancy between HbA1c and glucose levels, FPG and 2 h PG were considered more accurate [16]. Therefore, it may be beneficial for future research to consider both HbA1c and glucose levels when utilizing HbA1c as a diagnostic criterion, as this approach could potentially enhance specificity and reduce false-positive rates in diabetes diagnosis. In addition, missing data due to non-participation in follow-up, the deletion of outliers, and the negative values of the IGI_60_ and DI _IGI, ISI_ (11%) during log transformation for statistical analysis may have introduced unintended bias. However, the trajectories of the IGI_60_ and DI _IGI, ISI_ were similar to those of the HOMA-β and DI _HOMA-β, HOMA-IR_, suggesting that the elimination of negative values of the IGI_60_ and DI _IGI, ISI_ did not result in serious bias. Additionally, we used the IGI_60_ instead of the IGI_30_; however, the former is considered an acceptable measure to assess β-cell function [17,24]. Finally, limitations exist in interpreting the changes in β-cell function and insulin sensitivity around the menopausal period and their relationship with the risk of diabetes as a direct relationship between menopause and diabetes. These changes in β-cell function and insulin sensitivity indices may be closely related to the transition to diabetes. However, experimental studies have shown that reductions in estrogen and estrogen receptor activity are related not only to insulin resistance but also to β-cell survival and insulin secretion [42,43,44,45], potentially explaining the relationship between menopause and the increased risk of diabetes [46,47]. Therefore, future studies are needed to elucidate the relationship between menopause and changes in β-cell function and insulin sensitivity indices. For example, studies focusing on the connection among changes in female hormones directly associated with menopause, such as the estradiol/follicle-stimulating hormone, and β-cell function, insulin sensitivity indices, and the risk of diabetes will help to elucidate the relationship between menopause and the development of diabetes, as well as the related mechanisms.

## 5. Conclusions

This study investigated changes in the risk of diabetes, β-cell function, and insulin sensitivity during the menopausal phase, considering the timing of diabetes onset. The results showed that the risk of diabetes was higher in the pre- and perimenopausal periods than in the postmenopausal period, independent of age at menopause and obesity. Additionally, a decrease in insulin sensitivity followed by a subsequent decrease in β-cell function according to the time of onset was related to the risk of diabetes. We hope that this study expands the understanding of the risk of diabetes and related β-cell function and insulin sensitivity changes in the period around menopause, providing insights into the susceptibility to diabetes. Our findings highlight the need for health management and the prevention of diabetes among women undergoing menopausal transition. Specifically, healthcare providers might consider exploring early intervention strategies that focus on lifestyle modifications and the regular monitoring of metabolic indicators in this population. Additionally, it may be beneficial for public health policies to raise awareness and potentially provide resources for managing health risks during menopause. We believe that further studies should investigate the mechanisms involved in the onset of diabetes during the menopausal phase and the effect of the interaction between women’s predisposition to diabetes and menopausal changes on the risk of developing diabetes.

## Figures and Tables

**Table 1:** Baseline characteristics of women stratified into four groups.

		Non- Progressor to Diabetes (N = 996)		Women with Premenopausal Incident Diabetes (N = 36)		Women with Perimenopausal Incident Diabetes (N = 77)		Women with Postmenopausal Incident Diabetes (N = 115)		*p*-Value	Statistical Significance of Two-Groups Comparison (*p* < 0.05) *
		Mean n	SD %	Mean n	SD %	Mean n	SD %	Mean n	SD %		
Age (years)		44.1	3.0	42.7	2.3	44.5	3.1	44.9	2.8	0.001	a, d, e
Age at menopause (years)		50.8	2.7	51.7	2.5	51.3	2.6	50.2	2.8	0.007	e
Reproductive lifetime (years)		35.5	3.1	36.8	3.0	36.2	3.1	35.0	3.0	0.006	e
BMI (kg/m^2^) ^†^		23.9	1.1	25.3	1.1	25.6	1.1	25.4	1.1	<0.001	b, c
Waist circumference (cm) ^†^		76.5	1.1	78.7	1.1	80.3	1.1	80.4	1.1	<0.001	b, c
FPG (mg/dL)		79.8	6.6	85.9	11.7	86.6	8.9	83.3	7.8	<0.001	a, b, c, f
HbA1c		5.4	0.3	5.6	0.4	5.7	0.4	5.6	0.3	<0.001	a, b, c
Fasting insulin (μIU/mL) ^†^		6.5	1.8	7.4	1.8	7.2	1.9	8.2	1.5	0.001	c
IGI_60_^†^		7.3	3.9	5.3	2.3	4.8	4.3	7.4	3.0	0.065	
HOMA-β ^†^		153.1	2.0	136.3	2.0	119.4	2.0	160.3	1.8	0.011	b, f
Composite ISI ^†^		10.2	1.7	6.7	1.6	7.4	1.9	7.4	1.7	<0.001	a, b, c
HOMA-IR ^†^		1.34	1.8	1.56	1.9	1.53	2.0	1.68	1.6	<0.001	c
Disposition index _IGI, ISI_^†^		70.6	3.6	35.8	2.4	33.3	3.6	49.8	2.6	<0.001	a, b, c
Disposition index _HOMA-β,HOMA-IR_^†^		119.4	1.7	87.4	2.1	78.3	1.7	95.2	1.7	<0.001	a, b, c
Total cholesterol (mg/dL) ^†^		175.9	1.2	178.1	1.2	181.7	1.2	180.6	1.2	0.173	
HDL cholesterol (mg/dL) ^†^		46.5	1.2	43.2	1.2	43.4	1.2	43.0	1.3	<0.001	b, c
Triglycerides (mg/dL) ^†^		106.3	1.4	128.7	1.8	132.4	1.5	134.1	1.5	<0.001	b, c, d
Study Site (region)										0.278	
Ansan		618	62.0	28	77.8	47.0	61.0	70.0	60.9		
Ansung		378	38.0	8	22.2	30.0	39.0	45.0	39.1		
Education										0.256	
less than 7 years		166	16.7	4	11.1	14	18.4	28	24.6		
7~12 years		726	73.1	26	72.2	55	72.4	72	63.2		
more than 12 years		101	10.2	6	16.7	7	9.2	14	12.3		
Marital status										0.331	
single		8	0.8	1	2.8	1	1.3	1	0.9		
married		944	95.1	33	91.7	70	90.9	109	95.6		
divorced/separated/bereaved		41	4.1	2	5.6	6	7.8	4	3.5		
Income (Korean won)										0.364	
less than one million		163	16.8	7	19.4	17	22.7	22	19.5		
one to three million		581	60.0	16	44.4	40	53.3	68	60.2		
more than three million		225	23.2	13	36.1	18	24.0	23	20.4		
Family history of diabetes (yes)		232	23.3	15	41.7	27	35.1	36	31.3	0.004	a, b
History of GDM (yes)		10	1.0	1	2.8	1	1.3	0	0.0	0.332	
Hypertension (yes)		195	19.6	12	33.3	35	45.5	53	46.1	<0.001	b, c
Smoking											
never/former		953	98.0	30	88.2	73	96.1	108	97.3	0.003	a
current		19	2.0	4	11.8	3	3.9	3	2.7		
Exercise											
none		561	61.0	23	63.9	40	55.6	66	61.1	0.185	
one or two times per week		86	9.4	2	5.6	2	2.8	13	12.0		
three times or more per week		272	29.6	11	30.6	30	41.7	29	26.9		
Alcohol consumption											
none		657	67.9	18	51.4	52	68.4	73	64.6	0.003	a, b
less than 20 g per day		296	30.6	14	40.0	19	25.0	37	32.7		
20 g or more per day		14	1.4	3	8.6	5	6.6	3	2.7		

* comparison between groups: a = non- vs. premenopausal incident diabetes; b = non- vs. perimenopausal incident diabetes; c = non- vs. postmenopausal incident diabetes; d = pre- vs. perimenopausal incident diabetes; e = pre- vs. postmenopausal incident diabetes; f = peri- vs. postmenopausal incident diabetes. ^†^ log-transformed for statistical analysis and expressed as the geometric mean (geometric standard deviation). BMI, body mass index; FPG, fasting plasma glucose; HbA1c, hemoglobin A1c; IGI_60_, 60 min insulinogenic index; HOMA-β, homeostatic model assessment of β-cell function index; composite ISI, insulin sensitivity index; HOMA-IR, homeostatic model assessment of insulin resistance index; DI, disposition index; HDL, high-density lipoprotein cholesterol; GDM, gestational diabetes mellitus; SD, standard deviation.

**Table 2:** Odds ratios of diabetes development by the period relative to menopause.

	OR	(95% CI)	*p*-Value
Age at menopause	0.96	(0.90, 1.02)	0.210
Premenopausal aging	1.03	(1.02, 1.04)	<0.001
Perimenopausal aging	1.00	(0.99, 1.01)	0.862
Postmenopausal aging	0.96	(0.95, 0.97)	<.001
BMI *	6.89	(3.01, 15.82)	<.001
Waist circumference *	2.25	(1.41, 3.61)	0.001
HDL cholesterol *	1.00	(0.82, 1.23)	0.982
Triglycerides *	1.14	(1.08, 1.22)	<.001
Study site (region)			
Ansan	1.14	(0.77, 1.70)	0.515
Ansung	1 [Reference]		
Education			
more than 12 years	0.78	(0.38, 1.58)	0.484
7~12 years	0.89	(0.56, 1.39)	0.598
less than 7 years	1 [Reference]		
Marital status			
married	0.81	(0.17, 3.82)	0.791
divorced/separated/bereaved	1.52	(0.27, 8.66)	0.639
single	1 [Reference]		
Income (Korean won)			
more than three million	1.21	(0.68, 2.17)	0.515
one to three million	0.97	(0.60, 1.57)	0.897
less than one million	1 [Reference]		
Family history of diabetes (yes)	1.78	(1.23, 2.57)	0.002
Hypertension (yes)	2.99	(2.12, 4.21)	<.001
Smoking			
current	0.99	(0.63, 1.57)	0.978
never/former	1 [Reference]		
Exercise			
three times or more per week	0.99	(0.94, 1.04)	0.589
one or two times per week	0.95	(0.87, 1.04)	0.273
none	1 [Reference]		
Alcohol consumption			
20 g or more per day	1.02	(0.85, 1.21)	0.863
less than 20 g per day	0.99	(0.93, 1.04)	0.627
none	1 [Reference]		

* log-transformed for statistical analysis. OR, odds ratio; CI, confidence interval; BMI, body mass index; HDL, high-density lipoprotein.

**Table 3:** Odds ratio of diabetes development at baseline and longitudinal changes in the IGI_60_, composite ISI, and DI _IGI60, ISI_.

	Perimenopausal Incident Diabetes			Postmenopausal Incident Diabetes		
	OR	(95% CI)	*p*-Value	OR	(95% CI)	*p*-Value
	Model 1 *					
IGI_60_						
Baseline	0.86	(0.72, 1.02)	0.084	0.99	(0.83, 1.17)	0.893
Δ premenopausal period	1.16	(1.01, 1.32)	0.030	1.00	(0.82, 1.20)	0.962
Δ perimenopausal period	1.10	(1.01, 1.19)	0.031	1.02	(0.88, 1.17)	0.813
Δ postmenopausal period	-			1.12	(0.99, 1.26)	0.080
Composite ISI						
Baseline	0.65	(0.53, 0.80)	<0.001	0.77	(0.60, 0.98)	0.034
Δ premenopausal period	1.24	(1.02, 1.49)	0.027	1.33	(0.97, 1.81)	0.074
Δ perimenopausal period	1.01	(0.96, 1.06)	0.742	1.31	(1.03, 1.65)	0.027
Δ postmenopausal period	-			1.14	(0.89, 1.45)	0.295
DI _IGI60, ISI_						
Baseline	0.93	(0.89, 0.96)	<0.001	0.90	(0.86, 0.94)	<0.001
Δ premenopausal period	1.07	(1.03, 1.12)	0.002	1.10	(1.04, 1.16)	<0.001
Δ perimenopausal period	1.04	(1.01, 1.06)	0.007	1.09	(1.04, 1.14)	0.001
Δ postmenopausal period	-			1.08	(1.04, 1.13)	<0.001
	Model 2 *					
IGI_60_						
Baseline	0.85	(0.70, 1.03)	0.099	0.99	(0.84, 1.16)	0.870
Δ premenopausal period	1.17	(1.02, 1.33)	0.023	1.00	(0.83, 1.20)	0.987
Δ perimenopausal period	1.10	(1.01, 1.19)	0.030	1.02	(0.89, 1.17)	0.789
Δ postmenopausal period	-			1.12	(0.99, 1.26)	0.063
Composite ISI						
Baseline	0.60	(0.49, 0.74)	<0.001	0.77	(0.61, 0.98)	0.032
Δ premenopausal period	1.62	(1.28, 2.06)	<0.001	1.33	(0.98, 1.81)	0.072
Δ perimenopausal period	1.39	(1.13, 1.71)	0.002	1.30	(1.03, 1.65)	0.028
Δ postmenopausal period	-			1.14	(0.90, 1.45)	0.292
DI _IGI60, ISI_						
Baseline	0.92	(0.89, 0.95)	<0.001	0.90	(0.86, 0.94)	<0.001
Δ premenopausal period	1.08	(1.03, 1.12)	0.001	1.10	(1.04, 1.16)	<0.001
Δ perimenopausal period	1.04	(1.01, 1.07)	0.008	1.09	(1.04, 1.14)	0.001
Δ postmenopausal period	-			1.08	(1.04, 1.13)	<0.001
	Model 3 *					
IGI_60_						
Baseline	0.76	(0.59, 0.98)	0.031	1.04	(0.86, 1.25)	0.690
Δ premenopausal period	1.31	(1.03, 1.68)	0.029	1.14	(0.91, 1.43)	0.245
Δ perimenopausal period	1.17	(1.02, 1.33)	0.023	1.07	(0.89, 1.30)	0.467
Δ postmenopausal period	-			1.28	(1.07, 1.52)	0.006
Composite ISI						
Baseline	0.54	(0.42, 0.70)	<0.001	0.73	(0.59, 0.92)	0.007
Δ premenopausal period	1.88	(1.33, 2.67)	<0.001	1.37	(1.01, 1.86)	0.041
Δ perimenopausal period	1.61	(1.20, 2.16)	0.002	1.33	(1.04, 1.71)	0.024
Δ postmenopausal period	-			1.11	(0.84, 1.46)	0.477
DI _IGI60, ISI_						
Baseline	0.91	(0.87, 0.95)	<0.001	0.82	(0.72, 0.93)	0.003
Δ premenopausal period	1.06	(1.01, 1.12)	0.020	1.17	(1.06, 1.30)	0.003
Δ perimenopausal period	1.04	(1.01, 1.08)	0.025	1.17	(1.04, 1.32)	0.011
Δ postmenopausal period	-			1.14	(1.06, 1.24)	0.001

* adjustment: model 1 for baseline age; model 2 for baseline age, body mass index (BMI), and waist circumference; model 3 for baseline age, BMI, waist circumference, age at menopause, study site, education, marital status, income, family history of diabetes, hypertension, smoking, exercise, alcohol consumption, high-density lipoprotein cholesterol, and triglycerides. Δ, change during the period; IGI_60,_ 60-min insulinogenic index; ISI, insulin sensitivity index; DI _IGI60, ISI_, disposition index based on IGI_60_ and composite ISI; OR, odds ratio; CI, confidence interval.

## Data Availability

All available data generated or analyzed during this study are included in this published article. The original cohort data are not available due to the strict confidentiality guidelines and regulations of the National Institute of Health. Access to research data is permitted only by the designated researcher, and data sharing is strictly prohibited.

## Abbreviations

The following abbreviations are used in this manuscript:

ANOVA	Analysis of Variance
BMI	Body Mass Index
CIs	Confidence Intervals
DI	Disposition Index
DIHOMA-β, HOMA-IR	Disposition Index based on HOMA-β and HOMA-IR
DI _IGI60, ISI_	Disposition Index based on IGI_60_ and Composite ISI
FPG	Fasting Plasma Glucose
GEE	Generalized Estimating Equation
HbA1c	Glycated Hemoglobin A1c
HDL	High-Density Lipoprotein
HOMA-β	Homeostasis Model Assessment of β-cell function
HOMA-IR	Homeostasis Model Assessment of Insulin Resistance
HRT	Hormone Replacement Therapy
IGI60	60 min Insulinogenic Index
ISI	Insulin Sensitivity Index
KoGES	Korean Genome and Epidemiology Study
LMM	Linear Mixed Model
NIH	National Institute of Health
OGTT	Oral Glucose Tolerance Test
OR	Odds Ratio
PG	Plasma Glucose
SD	Standard Deviation
TC	Total Cholesterol
TG	Triglycerides
β-cell	Beta-cell
